# ELISA for detection of humoral immunity against measles

**DOI:** 10.17179/excli2015-607

**Published:** 2015-10-20

**Authors:** Muhammad Asif Zahoor, Muhammad Saqalein, Zeeshan Nawaz

**Affiliations:** 1 Department of Microbiology, Govt. College University, Faisalabad-Pakistan

## Dear Editor,

Measles is a highly infectious and contagious disease of the respiratory system caused by *Morbilivirus* (Hashiguchi et al., 2011[5]). The clinical disease has been characterized by high fever, cough, conjunctivitis, coryza, malaise, maculopapular rash and erythematous patches throughout the body along with increased mortality among non-vaccinated children (Ellison, 1931[3]; Fazlalipour et al., 2008[4]). Expanded Program on Immunization (EPI) has been launched in 1974 under the umbrella of World Health Organization (WHO) with the ultimate objective of elimination of Vaccine Preventable Diseases (VPDs) in children, further this program was initiated in Pakistan during 1978 (Ali, 2000[1]; Bugvi et al., 2014[2]). Among the VPDs, measles has been considered as one of most important diseases in children throughout the world particularly in developing countries (Rabenau et al., 2007[10]; Liu et al., 2013[7]). Furthermore, vaccination against measles is widespread which significantly reduces the mortality and morbidity across the globe (Perry and Halsey, 2004[9]; Leuridan and Van Damme, 2007[6]). Vaccination with first dose is recommended at the age of 9 months with booster dose at the age of 12 or 15 months with either live attenuated measles virus or Measles-Mumps-Rubella complex (MMR) as described (Leuridan and Van Damme, 2007[6]; Sheikh et al., 2011[11]). The immunization against measles virus usually resulted in protection due to humoral as well as cell mediated immune response (Ovsyannikova et al., 2003[8]). Many immunodiagnostic methods have been used for the detection of anti-measles antibodies as described by Perry and Halsey (2004[9]). Among those, Enzyme Linked Immunosorbent Assay (ELISA) has been reported as more sensitive and specific with reproducibility of results for detection of anti-measles antibodies (Fazlalipour et al., 2008[4]). Further, ELISA for measles has been reported to detect and quantify anti-measles IgG antibodies with high sensitivity and specificity (Rabenau et al., 2007[10]). 

Therefore, keeping in view the importance and significance of measles, this preliminary study was designed to develop an ELISA for detection of humoral immunity. For this purpose ELISA plates were coated with measles virus CAM-70 strain (PT Bio Farma, Bandung, Indonesia) which contain not less than 1000 CCID50 in 0.5 ml. Coating of plates was performed using carbonate buffer as described by van der Werff (2008[12]). Briefly, 100 µl of virus diluted with 0.05 M carbonate buffer was added in wells of ELISA plates in duplicate, following overnight incubation at 4 °C, blocking was achieved by adding 300 µl of blocking buffer (PBS + 10 % w/v skimmed milk powder). ELISA was performed as recently described by Zahoor et al. (2015[13]). Briefly, each serum sample was diluted with serum diluent and 100 µL of each diluted serum sample was added in each well of ELISA plate along with controls (Nova Tec Immunodiagnostica GmbH, Germany). Following incubation of ELISA plate for one hour at 37 °C, plate was washed with 300 µl of washing buffer. In the next step, 100 µl measles anti-IgG conjugate (Nova Tec Immunodiagnostica GmbH, Germany) was added into all wells except for the blank well, and kept for 30 minutes at room temperature. Finally, 100 µl tetramethylbenzidine substrate (TMB) was added into all wells, and kept at room temperature for 15 minutes in dark. Reaction was stopped by using 100 µl of stop solution into all wells. Absorbance of the ELISA plate was determined at 450 nm within 30 minutes after addition of the stop solution (Biotek®, ELX 808, USA). Altogether, it was concluded that the developed ELISA may be used to detect humoral immunity from vaccinated children to evaluate the effectiveness of measles vaccination following further standardization and optimization of developed ELISA protocol. 

## Conflict of interest

The authors declare that they have no conflict of interest.
